# Patterns of co-speciation and host switching in primate malaria parasites

**DOI:** 10.1186/1475-2875-8-110

**Published:** 2009-05-22

**Authors:** László Zsolt Garamszegi

**Affiliations:** 1 Department of Evolutionary Ecology, Estación Biológica de Doñana-CSIC, c/Americo Vespucio, s/n, 41092, Sevilla, Spain

## Abstract

**Background:**

The evolutionary history of many parasites is dependent on the evolution of their hosts, leading to an association between host and parasite phylogenies. However, frequent host switches across broad phylogenetic distances may weaken this close evolutionary link, especially when vectors are involved in parasites transmission, as is the case for malaria pathogens. Several studies suggested that the evolution of the primate-infective malaria lineages may be constrained by the phylogenetic relationships of their hosts, and that lateral switches between distantly related hosts may have been occurred. However, no systematic analysis has been quantified the degree of phylogenetic association between primates and their malaria parasites.

**Methods:**

Here phylogenetic approaches have been used to discriminate statistically between events due to co-divergence, duplication, extinction and host switches that can potentially cause historical association between *Plasmodium *parasites and their primate hosts. A Bayesian reconstruction of parasite phylogeny based on genetic information for six genes served as basis for the analyses, which could account for uncertainties about the evolutionary hypotheses of malaria parasites.

**Results:**

Related lineages of primate-infective *Plasmodium *tend to infect hosts within the same taxonomic family. Different analyses testing for congruence between host and parasite phylogenies unanimously revealed a significant association between the corresponding evolutionary trees. The most important factor that resulted in this association was host switching, but depending on the parasite phylogeny considered, co-speciation and duplication may have also played some additional role. Sorting seemed to be a relatively infrequent event, and can occur only under extreme co-evolutionary scenarios. The concordance between host and parasite phylogenies is heterogeneous: while the evolution of some malaria pathogens is strongly dependent on the phylogenetic history of their primate hosts, the congruent evolution is less emphasized for other parasite lineages (e.g. for human malaria parasites). Estimation of ancestral states of host use along the phylogenetic tree of parasites revealed that lateral transfers across distantly related hosts were likely to occur in several cases. Parasites cannot infect all available hosts, and they should preferentially infect hosts that provide a similar environment for reproduction. Marginally significant evidence suggested that there might be a consistent variation within host ranges in terms of physiology.

**Conclusion:**

The evolution of primate malarias is constrained by the phylogenetic associations of their hosts. Some parasites can preserve a great flexibility to infect hosts across a large phylogenetic distance, thus host switching can be an important factor in mediating host ranges observed in nature. Due to this inherent flexibility and the potential exposure to various vectors, the emergence of new malaria disease in primates including humans cannot be predicted from the phylogeny of parasites.

## Background

Parasites do not randomly infect hosts, as their reproduction needs to be adapted, at least to some degree, to the environment within the host. When hosts undergo certain selection regimes, their parasites might also take subsequent evolutionary steps to achieve maximal fitness in the new situation. As a result, parasites may be expected to co-evolve with their hosts. Parasites may also jump onto new hosts and start to exploit these as alternative resources, which may provide them with additional breeding opportunities. Alternatively, speciation may occur without host change, leading to the duplication of lineages within the same host. Finally, parasites may become extinct, if their evolution does not follow the diversification of hosts. Therefore, co-divergence (or co-speciation), horizontal transfer (or host switching), duplication and extinction (or sorting) are the key elements of the historical association between hosts and parasites [1].

Co-speciation has been demonstrated in several parasite systems, including lice, thrips, worms, viruses, bacterial and viral symbionts [[2-5], e.g. [6,7]]. The evolutionary history of malaria pathogens also provides examples co-evolutionary patterns. Phylogenetically related parasite species exploit phylogenetically related host species of vertebrates, and the division of bird-, reptile- and mammal-specific parasites delineates true taxonomic categories. This pattern at the broad scale may appear to be a result of co-speciation, but can actually be produced by evolutionary forces associated with vector shifts into different dipteran families [8]. The evolutionary routes within malaria parasites of birds and reptiles took two directions that apparently followed the evolutionary history of their hosts [9-11]. The ancestral species diverged and produced the genus *Haemoproteus *and *Plasmodium*, with the former displaying only sexual reproductive cycles in the hosts while the latter requiring asexually reproducing merozoite cycles before gametogenesis. The avian *Haemoproteus/Plasmodium *split was very close to the split between passerine and non-passerine hosts, thus the current diversity of avian malaria parasites has accompanied the radiation of modern birds. Hence, the evolution of malaria parasites in birds shows strong associations with host evolution and distribution, and has resulted in certain degree of conservativeness of host specificity [10,12,13]. However, this is not an exceptional case, as most of these major groups have wide host distributions [8]. There is ongoing debate about the phylogenetic history of primate-infective plasmodia [14], thus it remains an intriguing question as to whether the appearance of the major parasite lineages of this clade and their subsequent evolution were mediated by analogue changes in the corresponding host phylogeny [15].

Co-speciation alone is unlikely to shape the diversification and radiation of malaria parasites. The disease is spread by dipteran vectors that are generalist and thus easily establish parasite transmission across distantly related hosts, which facilitates host switches [9]. Moreover, vertebrate hosts are mobile, can migrate across large distances and often form flocks with other species or share habitats with diverse fauna [12,13,16], factors which all favour parasite transmission across species borders, and which weaken the phylogenetic association between host and parasite phylogenies. Hence, malaria parasites can disperse between sympatric host populations through host switches. Accordingly, lateral transfer between birds and reptiles may have occurred several times, while the utilization of mammals as hosts appears to have arisen via at least one host switch from a reptile host [11,17]. At a finer scale, some bird malarias are able to infect hosts across different families, and statistical evidence proves that current distribution of parasites may also reflect relatively frequent acquisition of new hosts by individual parasite lineages [9].

Frequent lateral transfer also seems to be a remarkable component of evolution in the primate-infective malaria lineage. For example, the four human-infective malaria species may have arisen independently during the phylogenetic history, not necessarily resulting from the close association with the evolution of human hosts. *Plasmodium ovale *is an ancient divergence from the main lineage and shares ancestors with *Hepatocystis *parasites [11]. *Plasmodium vivax *is a part of a recent species radiation and seems to have been derived about 100,000–50,000 years ago in south-east Asia, from a species that had exploited macaques and switched to *Homo sapiens*, or even to *Homo erectus *[18]. The origin of *Plasmodium malariae *is debated, as it can be grouped with all the three complexes (*falciparum/vivax/ovale *groups). However, *P. malariae *may exist for many million years, and is only distantly related to the other human parasites. One theory about the origin of *P. falciparum *postulates that its ancestor derived from avian malaria parasites via lateral transfer [19,20], while recent studies suggest that it diverged from the primate parasite lineage [15,17,21]. However, it is still an open question whether the human parasite originates, through a host switch, from an ape parasite or *vice versa*, or these parasites are resulted from the close co-evolution with human and chimpanzees, in which they followed the *Homo/Pan *separation without lateral transfer.

Concerning parasites of non-human primates, host switches are suspected in parasites of New World primates. *Plasmodium simium *is closely related to *P. vivax*, and it is likely that this parasite was originally a human parasite that switched host when entering the Americas [22]. Similarly, parasitism due to *Plasmodium brasilianum *of platyrrhine primates may have been acquired by contact with humans that served as a basis for the transfer of the sister taxa, *P. malariae*. The opposite scenario cannot be excluded, as humans may also have obtained *P. malariae *from animal reservoirs via lateral transfer [23]. Moreover, the phylogeny of Asian and African parasites includes several disagreements with the host phylogeny that may suggest several host switches across distantly related primates. *Plasmodium *parasites have been detected in only four of the fifteen Cercopithecidae genera. In addition, while macaques in Asia are intensively parasitized, malaria infection has never been described in the sympatric langur, proboscis monkey or doucs [15]. More conspicuously, the twenty species of the African *Cercoptithecus *genus seem parasite free, while the three species of *Cercocebus *harbour three kinds of malaria parasites. Therefore, *Plasmodium *species are unlikely to be able to infect all available primates via lateral transfer. Hence, some degree of conservativeness should affect the host choice of these parasites.

Previous studies regarding the evolutionary history of malaria parasites in the light of that of their hosts provide evidence for the occurrence of multiple host switches, but patterns of co-evolution are also suggested in some cases. However, observed associations between host/parasite phylogeny may be caused by extinction and re-colonization events in the past that are difficult to document with extant species. Moreover, it should be remembered that having no evidence for malaria parasitism in certain hosts may reflect inadequate sampling and not necessarily the non-existence of *Plasmodium *infection in these hosts. To make inferences about the degree of host phylogeny mediating the evolution of host choice in the parasite, statistical analyses are needed to control for these confounding factors. Host switches and co-speciation have been demonstrated quantitatively in birds [9,10], but such approaches have not applied yet to study host diversification in the primate system.

The aim of this paper is to investigate the association between the evolutionary history of primate malaria parasites and that of their hosts, and the evolutionary constraints of host specialization. First, mathematical formulas will be used to test whether the probability of co-occurrence of parasites with their hosts represents random host choice or taxonomically constrained host selection. Second, by comparing parasite and host phylogenies, the role of co-divergence, duplication, sorting and horizontal transfer in mediating host-parasite relationships will be quantified. Phylogenetic analyses will be performed to test for the significant association between the evolutionary tree of primate hosts and their malaria parasites. Third, by estimating ancestral states of host ranges, host utilization patterns along the phylogenetic history of *Plasmodium *parasites will be traced that may help identify host-switching events on the tree. Finally, the similarity in physiological environment across the host range of parasites will be examined. These analyses can be used to make inferences about consistent selection pressures that may act within hosts and shape host choice and the degree of specialization. Based on our current knowledge about the phylogenetic history of malaria parasites and their host, in general, one can predict to detect statistical support for co-speciation and host switching.

## Methods

### Phylogeny of parasites and their hosts

**Figure 1:**
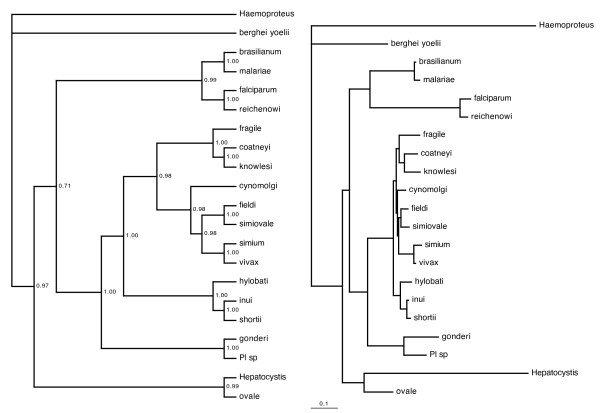
**Consensus phylogenetic hypotheses of *Plasmodium *parasites of primates from a Bayesian analysis of six genes**. Consensus tree that was obtained by adding sequences from rodent parasites and *Hepatocystis*. On the left panel, clade credibility trees are given with numbers at the nodes indicating the Bayesian posterior probabilities of each partition or clade in the tree, which are the proportion of trees in the sample that have the particular node. On the right panel, phylograms with branch lengths reflecting the expected substitutions per site is shown (see text for details).

The phylogenetic history of primate malaria parasites was reconstructed by using sequence information on six genes from various published sources. The combination of data from different studies was preferred, because different molecular markers gave different phylogenetic hypotheses [14], and it is important to account for such uncertainty in estimating phylogenetic relatedness. This can be achieved in a Bayesian framework, that incorporates Markov Chain Monte Carlo (MCMC) methods and provides a robust sample of alternative phylogenetic trees that can be fitted to the sequence data equally well [24,25]. The sequence information were gathered from the GenBank for the following genes: 18S rRNA for 15 species [15], beta-tubulin (nuclear gene), cell division cycle 2 (nuclear gene) and elongation factor Tu (plastid gene) for 10 species [18], cythocrome b (mitochondrial gene) for 14 species [17,21], and merozoite surface protein (nuclear gene) for 12 species [23,26-28]. Sequences were aligned as in the source papers by using MEGA. Altogether, it was possible to obtain aligned sequences for 18 *Plasmodium *species of primates. These were used to generate phylogenetic trees based on MCMC sampling in MrBayes 3.1 [25,29]. *Haemoproteus *was used as outgroup, while genetic data on other mammalian malaria species were also added (*Plasmodium berghei*, *Plasmodium yoelii *and *Hepatocystis*). Posterior distribution of trees were estimated from a Markov Chain implementing a general time reversible model of evolution with gamma correction for heterogeneity among sites (GTR + Γ model), which is widely used to model sequence evolution in primate malarias [e.g. [11,18,27]]. However, complex partitioned models were used, and all parameters were estimated separately for the individual genes. It was also assumed that the overall evolutionary rate was different across partitions. The chain used uniform prior probabilities on trees and the parameters of the model of sequence evolution, and an exponential prior on branch length. After reaching convergence, the chain was sampled 1,000 times at intervals of 10,000 trees (10 million iterations) after a stationary point (burn-in) that was identified based on (1) plots of log likelihoods over time, (2) similarity in topologies, branch support (posterior probabilities), and log likelihoods between trees from each replicate, and (3) average standard deviation of split frequencies between runs. This sampling provided an independent set of trees (serial autocorrelations: log-likelihood, r = 0.0005; tree-length, r = 0.0013, N = 1000). The phylogeny and branch lengths were estimated from the majority-rule consensus of the pooled post burn-in trees from the two replicates. The nodes of the consensus tree had very high (> 95%) Bayesian posterior probabilities indicating that these nodes were represented in almost the full proportion of trees in the sample (Figure 1). There was one exception close to the root of the consensus tree, which was supported only in the 71% of all trees. The removal of the genetic data for rodent malaria species and *Hepatocystis *from the MCMC sampling of phylogeny (with the same settings as above) provided alternative phylogenetic resolution at this node (Additional file 1). Hence, in the following analyses of historical association between hosts and parasites, both consensus trees representing two different resolutions at the uncertain node were used, and the corresponding results are provided in parallel. Figures for the former resolution are presented within the paper, while figures for the latter alternative resolution are given as additional files 1, 2 and 3. Phylogenetic methods based on Bayesian approaches are available to estimate ancestral states, and these can handle the whole sample of Bayesian trees and thus can take phylogenetic uncertainties into account. Accordingly, the complete sets of trees were used in these analyses.

The phylogeny of primate hosts is less debated. Phylogenetic reconstructions based on Bayesian analyses of *cytochrome-b *markers have been made for a subset of species [30], which agreed with the major taxonomic features identified in an earlier composite estimate based on parsimony algorithm to over a hundred trees [31]. Nonetheless, there was noticeable uncertainty about some nodes, emphasizing the need for a Bayesian approach that accounts for such phylogenetic uncertainty in the future. Currently, insufficient genetic information is available to create a complete Bayesian phylogeny for primates including more than 200 extant species. This reconstruction would require sequence data for the same genes sampled across the majority of species. Therefore, the phylogeny of host species was taken from the most recent primate supertree, whose topologies were derived from a set of partially overlapping, smaller source trees originating from previously published studies that had been based on a variety of data types, reconstructed using a variety of methods. This supertree was obtained from Bininda-Emonds *et al *[32], which is a complete species-level phylogeny of extant mammals from over 2,500 partial estimates, with divergence times estimated from genetic information in conjunction with cladistically robust fossil calibration points.

### The probability of co-occurrence of parasites and hosts

The list of host species of each parasite was obtained from reports on natural infections [15,23,33-39]. Based on this criteria *P. vivax *and *P. malariae *were considered to infect apes in addition to humans [[34], see also [40]]. Moreover, recent studies revealed that *Plasmodium knowlesi *is likely a recently emerged species in humans, in the process of becoming the 'fifth' human malaria parasite [41-44], which was also considered in the subsequent analyses of host – parasite relationships.

The lists of hosts were grouped into distinct clades (genus and family) to quantify the degree of host sharing. The probability approach of Ricklefs and Fallon [10] was used to test whether related parasite species tend to infect hosts within the same taxonomic clade or region. Since this method does not require phylogenetic information, all the 26 primate malaria parasite species that have been described taxonomically [34] were included. The probability of randomly drawing two host species from the same particular taxonomic group can be calculated as

(1)

where *N *is the total number of host species sampled worldwide, *N*_*i *_is the number of species in each *i *region, and *H*_(*i*, *j*) _is derived from

(2)

that gives the probability of detecting a pair of host species from the same taxonomic group *j *within each geographic region *i *[10]. The significance of *H *can be derived from binominal probability calculations.

### Historical association between primates and their malaria parasites

Several methods have been developed to study host and parasite co-evolution [2]. The majority of methods aim at reconstructing a putative history of host-parasite associations by mixing the four types of co-evolutionary events (co-speciation, switching, sorting and duplication) in a manner that provides the less costs of the estimated evolutionary scenario [1]. For the data at hand (hosts being infected by more than one parasite, and parasites infecting more than one host), the most suitable method was the algorithm that is implemented in program *TreeFitter *[45]. This approach relies on the event-based comparison of trees and uses parsimony criteria to estimate the number of co-divergence, duplication, sorting and switching events to match host-parasite phylogenies. The comparison uses the assignment of costs to each type of event to weight its probability of occurring. *TreeFitter *was unable to handle the large and incompletely resolved phylogeny of all extant primates (233 species) as provided by Bininda-Emonds *et al *[32]. Therefore, the tree was trimmed up to the genus level, and the remaining few polytomies were resolved at random, resulting in 60 tips. Hence, the association between parasite species and host genus was in the focus in *TreeFitter*.

Following the general practice [45], the *TreeFitter *analysis was started by using the default cost settings (0, 0, 1, 2; for co-divergence, duplication, sorting and switching, respectively). Given that this cost assignment is somewhat arbitrary, and the results may be sensitive to alternative settings, separate analyses with different cost structures are needed to assess the robustness of the results, and the relative contribution of each type of events. Therefore, the outcome of the following alternative cost settings was also assessed: (0, 1, 1, 1), as suggested by Page [46]; (-1, 0, 0, 0), the maximum co-divergence model [45]; (∞, 0, ∞, 1), Fitch optimization that excludes co-speciation and sorting via very high costs. Moreover, the overall fit and the contribution of different events were also estimated when cost structures varied among 0.5, 1.0 and 2 (maximum fourfold differences). Finally, the contribution of co-divergence, duplication, sorting and switching was investigated by preventing individual events one by one by the assignment of very high costs (100).

Inferences about the constrains of the congruent evolution between malaria parasites and their primate hosts or about the number of events can be tested against inferences drawn from random data sets in *TreeFitter*. Random trees are created from the original parasite and host trees by random permutation or Markov process. The significance of each combination of events considered was evaluated against the null hypothesis that such a historical scenario was statistically indistinguishable from a pattern arising from the randomization tests. Phylogenetic significance for different scenarios was determined based on the probability of observing a particular number of events within the 10,000 random permutations of the opposing phylogenetic trees.

**Figure 2:**
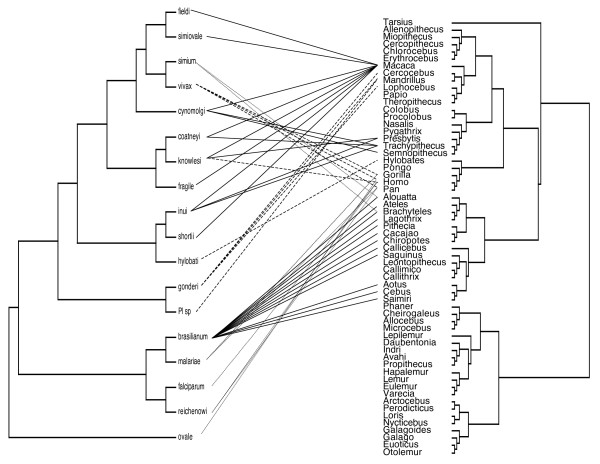
**The phylogenetic tree of the primate genus and their malaria parasite species**. *Parafit *outcome when using the parasite phylogeny from Figure 1. Connected taxa indicate naturally occurring infections. Solid lines represent host-parasite links that represent highly significant tendency for co-speciation, as shown by the *ParaFit *results. Dashed lines are for marginally (P ~0.1) significant relationships, while dotted lines indicate probabilities that correspond to random chance. Note that for simplicity, the phylogeny of hosts is not shown beyond the genus level, whereas the complete species-level phylogeny was used in the *ParaFit *analyses.

The software *ParaFit *follows a matrix approach to perform a global test of host-parasite co-speciation, but can also be used to assess the relative weight of each parasite-host link in mediating congruent evolution [47]. *ParaFit *was used to combine phylogenetic distance matrices of both the hosts and parasites and a matrix of incidences of infection (yes or no) between parasites and hosts, and then to compare this observed matrix with an expected matrix that can be calculated by the randomization of the incidence matrix. This approach was able to use phylogenies (with branch length and polytomies) describing relationships among species for both hosts and parasites. However, for illustrative purposes, associations between trees are presented by using the genus-level tree of primates (Figure 2 and Additional file 2).

### Ancestral host ranges

The Markov models of discrete character state evolution [48] were used to reconstruct the ancestral states of host use (i.e. to which degree hosts are taken from hominoids, cercopithecids or platyrrhine families) on the reconciled distribution of topologies by using *BayesTraits *[24]. A Markov chain was allowed to run for 101 million cycles after convergence, which was assessed by comparing results across five runs and plotting time series graphs. Rate coefficients and ancestral states probabilities were sampled every 10000 generations after a burn-in of 1,000,000 iterations. The transition rate parameters of continuous-time Markov models of trait evolution were conditioned by adjusting the *ratedev *parameter to a value that provides an acceptance rate of newly proposed states of the rate parameters between 20 and 40%. To assess the robustness of model convergence, five independent Markov chains of 101,000,000 observations were run, and all of these observations converged on similar direction providing very similar posterior distributions of rate parameters and ancestral state estimates. The sampling of the chains (at each 10,000^th ^iteration after burn in) resulted in a sample of 10000 observations, which can be considered independent (serial autocorrelation based on log-likelihood, r = 0.046, N = 10000). Extremely long runs (1,001,000,000) with a sampling at longer intervals (100,000) lead to an increase in independence between samples (serial autocorrelation based on log-likelihood, r = -0.0001, N = 10000), but the results (rate parameters and ancestral states) were basically identical to what could be obtained with sorter runs. Therefore, results are calculated based on samples that relied on 101,000,000 iterations.

As the posterior results from a Markov chain may be sensitive to prior distributions, hyperpriors were used, that seed priors from a uniform distribution on a given interval and distribution (gamma or exponential), and thus reduce some of the uncertainty and arbitrariness of choosing priors. Having no *a priori *assumptions about the shape of parameter distribution, gamma distributions were applied generally, which can take a variety of uni-modal shapes or even mimic exponential distribution (the use of beta distributions was avoided, as beta priors are not yet fully implemented in *BayesTraits*). The intervals of the mean and variance of the prior distribution were set by using information from the Maximum Likelihood results obtained for all phylogenetic hypotheses considered (1000 trees corresponding to 1000 models of trait evolution). These prior settings yielded posteriors that produced good fits of the data to the model of trait evolution without imposing unrealistic restrictions on the rates (i.e. they allowed wide posterior distributions of the rate parameters and resulted the highest log-likelihood).

### Consistent selection pressures across hosts

Parasites can be hypothesized to non-randomly infect all available hosts, because they can infect hosts that provide a similar environment only. As erythrocytes represent the main environment for malaria parasites [49], it can be predicted that hosts infected by the same parasite species would display similar erythrocyte characteristics. Particularly, the availability of resources within the host body for the parasite may be a function of erythrocyte size. The volume of the red blood cell can create constraints for the within-cell growth during the merozoite phase, as larger host cells could provide more space and supply than smaller cells. Therefore, if a parasite species is selected to match its reproductive features with a certain erythrocyte size in a certain host (e.g. by particular periodicity or merozoite number), after a host switch, it will be more likely established in a new host that has similar erythrocyte characteristics. As a result the observed host range of parasites should be composed of host species with comparable erythrocyte size. To test whether erythrocyte size varies systematically within the hosts of parasites, mean corpuscular volume (MCV) was used that corresponds to a wet volume in μm^3 ^(or fL) as provided by the Genome Size Database [[50], ]. MCV is usually measured directly with a Coulter counter, but can also be calculated as the ratio between haematocrit and red blood cell count. The use of information on wet volume over dry volume was preferred, as the former is more directly reflects the constraint on parasite growth. For some host species, that were not listed in the Genome Size Database, information on cell volume were derived from other sources [[51-54] and International Species Information System, Minnesota Zoological Garden, Apple Valley, MN]. Data on host body size were used from Smith and Leigh [55] to control for the potentially confounding effect of allometric scaling of erythrocyte traits [56] that deserves appropriate statistical treatment. In addition, host specialization may be directly related to life history traits as reflected by body size [57-59], that should also be controlled for.

By including the available information on different hosts of *Plasmodium *parasites, the gathered data were tabulated at the host level. Each host entry corresponded to a primate species that is a known natural host of the parasite. If a given host is infected by more than one parasite, a multiple entry for that host species was created, which were thus associated with different *Plasmodium *species. To assess the degree to which parasites are selected by host erythrocyte size, it was tested whether the variation of this variable was larger between than within *Plasmodium *species, while controlling for host species identity as a random factor and host body mass as a fixed effect in a mixed generalized linear model.

## Results

### The probability of co-occurrence of parasites and hosts

There are 26 taxonomically described primate malaria parasites, which can be detected in 53 host species spanning across 25 genus and 8 families. This distribution represents 93 host-parasite relationships. Nine *Plasmodium *species appear very host specific, as they can be observed only in a single host species. More generalist parasites can occupy from two up to 27 primate hosts (mean ± SE species-specific host range including specialists: 3.62 ± 1.05 with median = 2), with *P. brasilianum *representing the most extreme breadth of host range (Figure 2 and Additional file 2). These host ranges at the species level cover 1–12 taxonomic groups at the genus level (mean ± SE genus-specific host range: 2.11 ± 0.42 with median = 2), while host ranges usually tend to fall within the same family, except in the cases of *P. brasilianum *and *P. knowlesi*, which infect hosts from three and two families, respectively.

Among the nine parasite species recovered from exactly two hosts, only two of the host pairs derived from the same genus. The remaining seven host pairs came from closely related genus within the same family. Using equations (1–2) to calculate the probability of drawing two hosts from the same taxon gives *H *= 0.107 when the genus level is considered as the focal taxonomic unit, and geographic regions are discriminated at a large scale (Africa, Asia, South-America and Madagascar). This corresponds to a statistical significance of *P *= 0.187 (two-tailed), when the interest is to calculate the probability of finding exactly two of nine pairs of hosts in the same genus. This significance value indicates that it is not possible to reject the hypothesis that random chance alone can create the observed patterns at the genus level. Hence, primate malaria parasites do not seem to link very tightly to specific host genus once they are able to infect at least two hosts.

On the other hand, at the family level, there seems to be a close association between host specificity and host taxonomy across different geographic regions. A random choice would raise a probability of *H *= 0.420 to detect host pairs within the same family and geographic region. Among the 17 generalist parasites, all but two choose host from the same family, which strongly, at P = 0.007 significance level, deviates from being accidental.

### Historical association between primates and their malaria parasites

The analysis using the default cost settings in *TreeFitter *(0, 0, 1, 2; for co-divergence, duplication, sorting and switching, respectively) suggests that there is a phylogenetic association between parasite species and host genus, as illustrated in Figure 2 and Additional file 2. The permutation tests revealed that fitting host and parasite trees results in significantly lower cost structure than it could be expected by chance independent of the parasite tree considered (Table 1). The reconstructions showed two to four co-divergences, eight to nine duplications, one to three sorting events and five to six switching events, depending on the phylogenetic hypothesis of parasites. The most consistent pattern emerged in the two analyses was that switching event was less likely than in the randomized trees, while one parasite phylogeny resulted in a co-divergence cost structure that occurred more frequently than random chance.

**Table 1:** The results of the *TreeFitter *analysis under various cost settings and under two different evolutionary hypotheses of primate malaria parasites

Using parasite phylogeny from Figure 1								
Event cost settings^a^				Cost	Co-divergence	Duplication	Sorting	Switching

0	0	1	2	13^cc^	2–3	8–9	1–3	5–6^c^
0	1	1	1	13^c^	4–4	1–2	0-0	10–12
-1	0	0	0	-8	8-8	1–9	21–93^c^	0–8
∞	0	∞	1	7^c^	0-0	10-10^b^	0-0	7-7^c^
0	0	0.5	2	11.5^cc^	3-3	9-9	3-3	5-5
0	0	1	1	7^c^	0–3	8–10^b^	0–1	6–7^c^
0	0	2	0.5	3.5^c^	0–2	8–10^b^	0-0	7-7^c^
0	0	2	1	7^c^	0–2	8–10^b^	0-0	7-7^c^
100	0	1	2	14^c^	0-0	10-10^b^	0-0	7-7^c^
0	100	1	2	124	4-4	1-1	0-0	12-12
0	0	100	2	14^c^	0–2	8–10^b^	0-0	7-7^c^
0	0	1	100	51	5-5	12-12	51-51	0-0

Using parasite phylogeny from Additional File 1								

Event cost settings^a^				Cost	Co-divergence	Duplication	Sorting	Switching

0	0	1	2	11^ccc^	4-4^b^	8-8	1-1	5-5^cc^
0	1	1	1	12^cc^	5-5	1–2	0-0	10–11
-1	0	0	0	-8	8-8^c^	1–9	20–93^c^	0–8
∞	0	∞	1	7^c^	0-0	10-10^b^	0-0	7-7^c^
0	0	0.5	2	10.5^ccc^	4-4	8-8	1-1	5-5
0	0	1	1	6^cc^	2–4^b^	8–9	0–1	5–6^cc^
0	0	2	0.5	3^cc^	2–3^b^	8–9	0-0	6-6^cc^
0	0	2	1	6^cc^	2–3^b^	8–9	0-0	6-6^cc^
100	0	1	2	14^c^	0-0	10-10^b^	0-0	7-7^c^
0	100	1	2	122	5-5	1-1	0-0	11-11
0	0	100	2	12^cc^	2–3^b^	8–9	0-0	6-6^cc^
0	0	1	100	51^c^	5-5	12-12	51-51^c^	0-0

The tests were performed between the consensus tree of primate malaria parasites given in Figure 1 and Additional File 1, and the phylogeny of primate host genus in Figure 2. For each model, overall costs and the reconstructed number of events (expressed as ranges that result in equal total costs) are given.

^a^Event costs are co-divergence (co-speciation), duplication (within-host speciation), sorting (extinction) and switching (host change via lateral transfer), respectively.

^b^Overall cost/the number of events significantly exceeds that for randomized trees (^b^P < 0.05, ^bb^P < 0.01, ^bbb^P < 0.001).

^c^Overall cost/the number of events is significantly less than that for randomized trees (^c^P < 0.05, ^cc^P < 0.01, ^ccc^P < 0.001).

Subsequent analyses exploiting different cost assignments are also summarized in Table 1. An alternative cost setting reducing the penalty on switching (0, 1, 1, 1) also showed significant fit between host and parasite trees. However, the contribution of events to this fit was modified, as the number of duplication events declined due to the costs applied, while the number of host switching events increased. Applying maximum co-divergence (-1, 0, 0, 0), the overall cost of the model was very low, and a large number of extinction was required to balance the predominance of co-speciation. Fitch optimization that excludes co-speciation and sorting via very high costs (8, 0, 8, 1) returns with a significant relationship between host and parasite phylogenies, which can be achieved if a large number of duplications and switches are allowed. When sorting and switching costs varied among 0.5, 1.0 and 2, the overall fit was still significant, with a strong contribution of switching. Cost structures for duplication and co-divergence demonstrated that these events could also play some roles in mediating the association between host and parasite trees depending on the evolutionary scenario considered for parasites. Preventing individual events one by one by the assignment of very high costs (100) returned with significant correlations between the two phylogenies, except when duplication and switching are prevented. This may suggest that the exclusion of these events considerably reduces the probability of finding correlated evolution.

Altogether, there seems to be a strong association between parasite and host phylogenies. The main factor contributing to this fit is the relatively small number of switching events, and depending on the parasite phylogeny used, the large numbers of co-speciation or duplication event. Sorting can play a role only under some extreme scenarios, when it is to balance the high degree of co-divergence or the low degree of switching.

In the application of *ParaFit *to the phylogenetic data, the global test of association also provided significant evidence for congruent evolution (using parasite phylogeny from Figure 1: ParaFitGlobal = 530560.47, P = 0.001, using parasite phylogeny from Additional file 1: ParaFitGlobal = 526811.09, P = 0.001) indicating strong phylogenetic structure behind the known host-parasite associations. However, the close inspection of individual parasite-host links suggests that not all *Plasmodium*-primate associations contribute equally to the global fit between the two data sets. When using parasite phylogeny from Figure 1, of 79 cases of infection, 59 (74.7%) had probabilities of < 0.05 and an additional 13 links (16.5%) were marginally significant (P ~0.1) (Figure 2). Similar patterns emerged, when an alternative resolution was used for parasite phylogeny (Additional file 1), with the difference that some associations became stronger (Additional file 2). Parasite clades, for which divergence follows that of their host, were those that infect South Asian cercopithecids, while there is also a remarkable association between *P. brasilianum *and its hosts. Interestingly, parasites of apes and humans are not so closely associated with their hosts.

### Ancestral host ranges

**Figure 3:**
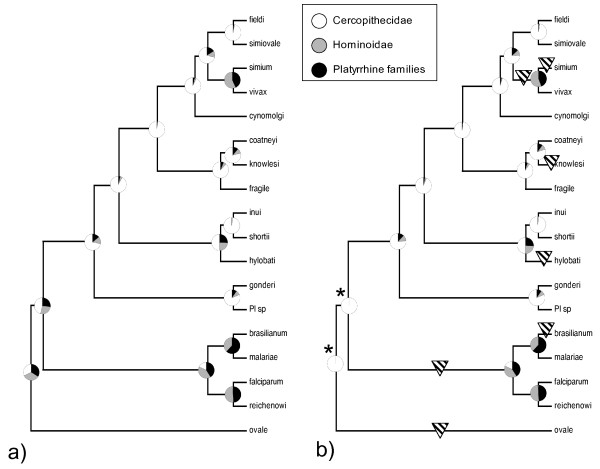
**Estimated ancestral states of host utilization based on Markov Chain Monte Carlo evolutionary modelling that used 1000 phylogenetic hypotheses**. Circles summarize posterior densities of the reconstructed ancestral state from the Markov chain of 101,000,000 independent evolutionary models. Pie charts present probabilities of hosts being hominoid, cercopithecid or platyrrhine primate, respectively. a): Ancestral state estimations, when no restrictions were made, and each node was allowed to take any of the tree states. b): Estimations, when information on fossil records (hominoids were unlikely to be present for parasites to infect around the origin of primate malarias) was used, and the two deepest nodes (marked with asterisk) were forced to have zero probability for hominoid host use. Results obtained when using the Bayesian sample of phylogenetic trees of parasites summarized on Figure 1. Triangles show branches where host switch across large phylogenetic distances should have occurred.

The reconstructions of ancestral states of host use along the phylogeny of primate malaria species based on Bayesian approaches are summarized in Figure 3 and Additional File 3. Without making any restrictions (i.e. parasites are equally allowed to infect cercopithecid, hominoid and platyrrhine hosts at each node), the posterior probabilities at the root of the tree indicated that the ancestor of recent *Plasmodium *species of primates could have used hosts from any large taxonomic group of primates (Figure 3a and Additional File 3a). However, during the subsequent evolution, at least one early split may have occurred, and one clade specialized on hominoids or platyrrhines, while another infected cercopithecids. Moreover, independent of the parasite phylogeny considered, there are clear indications for more recent host changes (e.g. *Plasmodium hylobati*, *P. vivax*, *P. simium *may have been resulted from switches from cercopithecid hosts, while *brasilianum/malariae *and *falciparium/reichenowi *pairs may have been also resulted from lateral transfer across large phylogenetic distances).

When considering time constraints, it is unlikely that the ancestor of primate parasites was confronted with hominoid hosts. Hominoids diverged only c.a. 20 Myr ago [32], while the origin of primate plasmodia can be dated much earlier [60]. Therefore, this information could be used as a fossil record. Accordingly, the two most distant nodes (root and the node below) were restricted to allow hosts from ancient primate hosts only (cercopithecids or platyrrhines). This modeling yielded high posterior probabilities for the ancestral parasite being a cercopithecid specialist species (Figure 3b and Additional File 3b). Subsequent evolutionary patterns of host use were similar to those provided by the previous models applying no restriction on any node. Therefore, one can identify, depending on the phylogeny used, at least seven to eight events that correspond to host changes across distantly related host taxa (Figure 3b and Additional File 3b).

### Consistent selection pressures across hosts

There was only a marginally significant evidence for wet erythrocyte volume being similar across the host range of parasites in a mixed model, in which hosts effects were controlled as random factor (F_25,9 _= 2.040, P = 0.133). This was also the conclusion from the statistical model that held constant allometric effect by including host body mass as a co-variate (parasite-specific effects: F_25,8 _= 2.882, P = 0.062; host body mass: F_1,8 _= 11.901, P = 0.009).

## Discussion

Various approaches were applied to estimate the relationship between the phylogenetic trees of primate malaria parasites and their hosts. These analyses unanimously suggested that the evolution of the focal parasites is constrained by the phylogenetic relatedness of their hosts, and that host switches play central role in shaping the distribution of malaria species across their primate hosts. First, parasites cannot randomly infect all geographically available primates, because they appear only to be able to fit their reproduction to closely related hosts that belong to the same family (the extremely generalist *P. brasilianum*, and *P. knowlesi *that has recently established natural infection in human should be considered as exceptions). Second, event-based reconciliation of parasites to host trees suggests that host switching is the most obvious event that shapes the association between host-parasites phylogenies, but depending on the parasite phylogeny considered, the role of either co-speciation or duplication cannot be generally excluded. Moreover, the contribution of particular host-parasite associations in mediating concordance between trees is different, as some links appear to represent stronger phylogenetic structure than others. Third, the reconstruction of ancestral states of host use revealed that several switches of hosts across large phylogenetic distances might have occurred during the long evolution of primate malarias.

Patterns of co-occurrence of primate malaria parasites and hosts were comparable to those that have been observed in avian plasmodia at the family and the clade levels [9,10]. Therefore, malaria parasites of vertebrates are generally conservative, and do not infect all potential hosts across wide phylogenetic distances. Only a single, highly generalist parasite, *P. brasilianum *is able to infect a large number of hosts, spanning three primate families (Aotidae, Atelidae, Cebidae). The evolutionary success of this species in various hosts may result from the fact that it is able to transmit via several vector species and from the absence of other competitor parasites in South America [34]. Moreover, it is also possible that the morphospecies *P. brasilianum *consists of several genetically distinguishable lineages that specialize on different hosts.

Malaria parasites usually have a host range that is constrained by their geographic distribution. Primate hosts are not randomly distributed across the globe, as certain taxonomic groups are associated with certain localities (i.e. prosimians in Madagascar, platyrrhins in South America, and catarrhines in Africa and Asia). Hence, malaria parasites found on different continents and countries are given a choice to infect hosts that are phylogenetically related to each other. Moreover, these parasites do not randomly infect all available primate hosts, thus they seem to have limited ability to parasitize on all sympatric primate populations. This non-random selection of hosts may imply that *Plasmodium *species are constrained to adapt to hosts that provide similar growth environments. Given that the environment for the parasites is established by the physiological characteristics of primate hosts that are strongly determined by their phylogenetic relationships, it was predicted that the host range of parasites covers sister species that constitute the most optimal conditions for reproduction. There was a non-significant tendency for that parasites – by infecting geographically and evolutionarily related species – are faced with consistent selective pressures, as variation in host erythrocyte size tended to be smaller within the host range of parasites than across hosts of different parasites. Based on the current data of limited availability, therefore, it would be immature to exclude the scenario that each *Plasmodium *parasite has to cope with a more or less consistent haematological profile when adapting to a certain range of hosts. However, other factors should also be considered, as various physiological and life history characteristics can affect the host choice of malaria parasites. Accordingly, similarity in the host environment can be manifested across multiple host traits, which warrants further investigation. In any case, the range of natural hosts should reflect the outcome of a long evolutionary adaptation and thus the role of specialization that is dependent on host phylogeny.

Further analyses demonstrated that host ranges of primate malarias observed today in nature resulted from long evolutionary history of adaptation, which is shaped by host switches. Studies on various host – parasite model systems have repeatedly demonstrated that co-evolution plays a key role in shaping the resemblance of parasite phylogenetic trees to that of their hosts, while host switching remains relatively infrequent in other parasites [e.g. [61-64]]. Malaria pathogens differ from ectoparasitic or bacterial parasites by being transmitted by dipteran vectors, which enhance lateral transfer between distantly related hosts and thus increase the probability of host switching [10]. Accordingly, several host switching events can be reconstructed on the available phylogeny of primate plasmodia.

For example, there may have been an early split of cercopithecid and hominoid/platyrrhine malarias. The cercopithecid line may have successfully radiated first in Africa in ancient mandrills, and later, a more recent adaptive radiation may have occurred in Asia in ancient macaques. The presence of suitable vectors and divergent host populations are generally envisaged as the responsible factors that allowed the successful spread of parasites in this region [33,65]. Moreover, even within the apparently host-conservative cercopithecid line, there are several examples of lateral transfer of malaria across hosts from different families (cercopithecid-hominoid host switch) (Figure 3 and Additional File 3). For example, the ancestor of the gibbon malaria, *P. hylobati *derived from *Plasmodium inui*-like parasite, whereas the human *P. vivax *also originates from a macaque parasite. In addition, the recently gained ability of *P. knowlesi *to grow in human hosts reflects the host switch of a parasite that primarily evolved to infect cercopithecid species. Furthermore, other recent nodes also involve flexible host ranges, as the ancestors of malarias infecting platyrrhine primates were probably omnipotent parasites with the capacity to grow in hominoids.

Parasites of the cercopithecid lineage demonstrate noticeable diversity in terms of life history, as their host specialization does not inevitably require the development of exclusive reproductive strategies that would restrict their potential to exploit alternative hosts. This group includes both relapsing and non-relapsing parasites, spans quartan, quotidian and tertian species and is basically represented by generalist plasmodia that can grow in up to eleven primate hosts (mean ± SE host range: 4.0 ± 0.99 species). A great diversity in fundamental reproductive strategies has thus been preserved. Such diversity can maintain the genetic variation on an evolutionary time scale, and can underlie successful host switch once conditions for transmission are fulfilled. Additionally, the associated anopheline vector populations deliver repeated chances of contacts with diverse primate fauna providing frequent opportunities for parasite transfer [34,66]. Macaque malarias rely on mosquitoes from the *Leucosphyrus *group that feed on various primates across broad taxonomic ranges including humans and orang-utans.

The results yield some additional details concerning the evolution of human malarias. For example, the current resolution suggests that the ascendant of *P. falciparum *and *Plasmodium reichenowi *may have involved species that could parasitize the ancestors of New World primates. This may imply that i) in the past, very generalist parasites may have existed that could realize host switches across large distances; or ii) that parasite lines followed the evolutionary splits of their hosts [21]. Unfortunately, the current results cannot bring us closer to the events that occurred *within *the *falciparum/reichenowi *line. It thus remains plausible that the recent host ranges of these *Plasmodium *species derive from a long-term co-evolution with the host species after a very early split, or from more recent switches between ape and human hosts. Moreover, the estimated ancestral states are consistent with both the anthroponosis and zoonosis theories of origin that may have been operated in South-America [22]. It is equally likely that the ancestors of the simian parasites (*P. brasilianum *and *P. simium*) were human malaria species (*P. malariae *and *P. vivax*), or *vice versa*. In order to understand the origin of New World malarias, we have to rely on the interpretations of events in association with temporal and spatial constraints [18,60]. This approach suggests, at least in the case of the *simium/vivax *pair that the human malaria *P. vivax *originates from a *Plasmodium *species infecting Asian cercopithecids that was subsequently brought to the New World and colonized simian primates giving birth to *P. simium*. Besides the unresolved uncertainties, one striking pattern emerges from the *ParaFit *results: the evolution of human malarias is not structured by host phylogeny, and it is likely that the history of human parasites involve host switches across large phylogenetic distances (Figure 3). Consequently, the emergence of a new human pathogen cannot be predicted from the parasite phylogeny. This is likely to be explained by the preserved flexibility of malaria parasites to infect alternative hosts, and the potential to use various vector species.

Host specialization can involve some costs, as long-term commitment to a particular host may reduce the genetic variation needed for the use of alternative hosts. Therefore, extreme specialization may represent an evolutionary-dead-end, in which further evolution via host switching is limited [67]. Accordingly, host-specific parasite lineages should be established during the early evolutionary history of the lineage, and such ancestral specialization should determine subsequent host diversification by constraining the frequency of host switches across wide phylogenetic distances [17]. The deterministic evolution of host ranges is evident from the phylogenetic history of *Plasmodium *species at a broad scale, as the specialization to the major vertebrate host groups has an ancient origin and subsequent host switching occurred rarely [11]. However, analyses within the avian parasite clade showed that host switching occurs relatively frequently even at the subterminal nodes of the parasite phylogeny, although these switches bridge closely related hosts [10]. On the other hand, the primate malaria system demonstrates clear host switches across large phylogenetic distances at both shallow and deep nodes of the phylogeny (Figure 3 and Additional File 3). This indicates that host specialization does not necessarily lead to evolutionary-dead-ends at a fine scale, and that the emergence of new diseases via lateral transfer across distantly related primate hosts cannot be ruled out.

Inferences about evolutionary events of parasites in the light of host phylogenies are sensitive to the phylogenetic information at hand. Although Bayesian approaches were used to deal with phylogenetic uncertainties, for some species no genetic data were available making it impossible to place them on the phylogenetic tree of primate malarias. Most importantly, information about the phylogenetic position of lemur (e.g. *Plasmodium girardi*) and hominoid (e.g. *Plasmodium rodhaini, Plasmodium schwetzi *and *Plasmodium pitheci*) parasites is lacking. Moreover, it might be that due to incomplete sampling, some lineages have not yet been discovered. By adding these missing or newly discovered parasite species to the parasite tree our picture about the details of co-speciation or host switching may change. To quantify the risk of the emergence of new malaria disease that may threaten humans or species with great concern, it would be crucial to test whether the remaining hominoid parasites are grouped together with the *falciparum/reichenowi *clade, or are spread throughout the phylogenetic tree. From such information, the likelihood of host switches between hosts closely related to humans could be estimated. However, from an evolutionary point of view, existing data already show strong evidence for congruence between host and parasite phylogenies and that host switching plays a key role in the evolution of malaria parasites of primates including those that infect humans.

## Conclusion

The systematic analysis of patterns of host-parasite co-evolution in the primate malaria system reveals that the phylogenetic history of primate-infecting plasmodia is constrained by the phylogenetic associations of their hosts. One of the most important factors that shape this pattern is hosts switching, and some parasites can preserve a great flexibility to infect hosts across a large phylogenetic distance resulting in broad host ranges that can be observed in nature. The emergence of new malaria disease in primates including humans cannot be fully predicted from the phylogeny of parasites.

## Competing interests

The author declares that they have no competing interests.

## Authors' contributions

LZG conceived of the study, collected data, designed and performed the statistical analysis, and drafted the manuscript.

## Supplementary Material

Additional file 1 **Consensus phylogenetic hypotheses of *Plasmodium *parasites of primates from a Bayesian analysis of six genes**. Consensus tree from a second modeling of phylogenetic relationships that excluded rodent parasites and *Hepatocystis *and provided alternative resolutions at the root. On the left panel, clade credibility trees are given with numbers at the nodes indicating the Bayesian posterior probabilities of each partition or clade in the tree, which are the proportion of trees in the sample that have the particular node. On the right panel, phylograms with branch lengths reflecting the expected substitutions per site is shown (see text for details). Click here for file

Additional file 2 **The phylogenetic tree of the primate genera and their malaria parasite species**. *Parafit *outcome when using the parasite phylogeny from Additional file 1. Connected taxa indicate naturally occurring infections. Solid lines represent host-parasite links that represent highly significant tendency for co-speciation, as shown by the *ParaFit *results. Dashed lines are for marginally (P ~0.1) significant relationships, while dotted lines indicate probabilities that correspond to random chance. Note that for simplicity, the phylogeny of hosts is not shown beyond the genus level, whereas the complete species-level phylogeny was used in the *ParaFit *analyses. Click here for file

Additional file 3 **Estimated ancestral states of host utilization based on Markov Chain Monte Carlo evolutionary modelling that used 1000 phylogenetic hypotheses**. Circles summarize posterior densities of the reconstructed ancestral state from the Markov chain of 101,000,000 independent evolutionary models. Pie charts present probabilities of hosts being hominoid, cercopithecid or platyrrhine primate, respectively. a): Ancestral state estimations, when no restrictions were made, and each node was allowed to take any of the tree states. b): Estimations, when information on fossil records (Hominoids were unlikely to be present for parasites to infect around the origin of primate malarias) was used, and the two deepest nodes (marked with asterisk) were forced to have zero probability for hominoid host use. Results obtained when using the Bayesian sample of phylogenetic trees of parasites summarized on Additional file 1. Triangles show branches where host switch across large phylogenetic distances should have occurred. Click here for file
