# Supplementary figures and images for: Patterns of co-speciation and host switching in primate malaria parasites
Source: Malar J. 2009 May 22;8:110. doi: 10.1186/1475-2875-8-110 (PMC2689253; doi:10.1186/1475-2875-8-110)

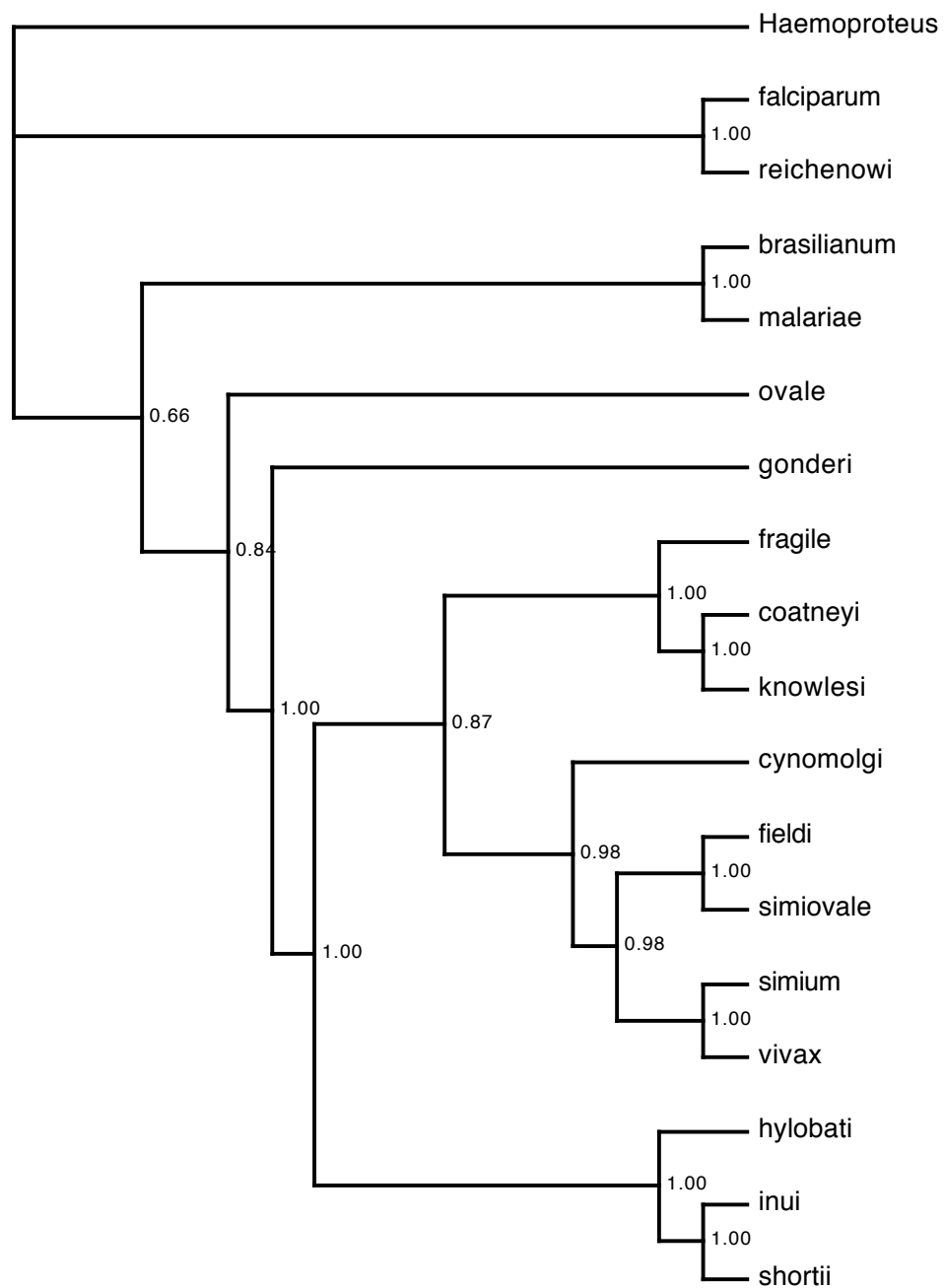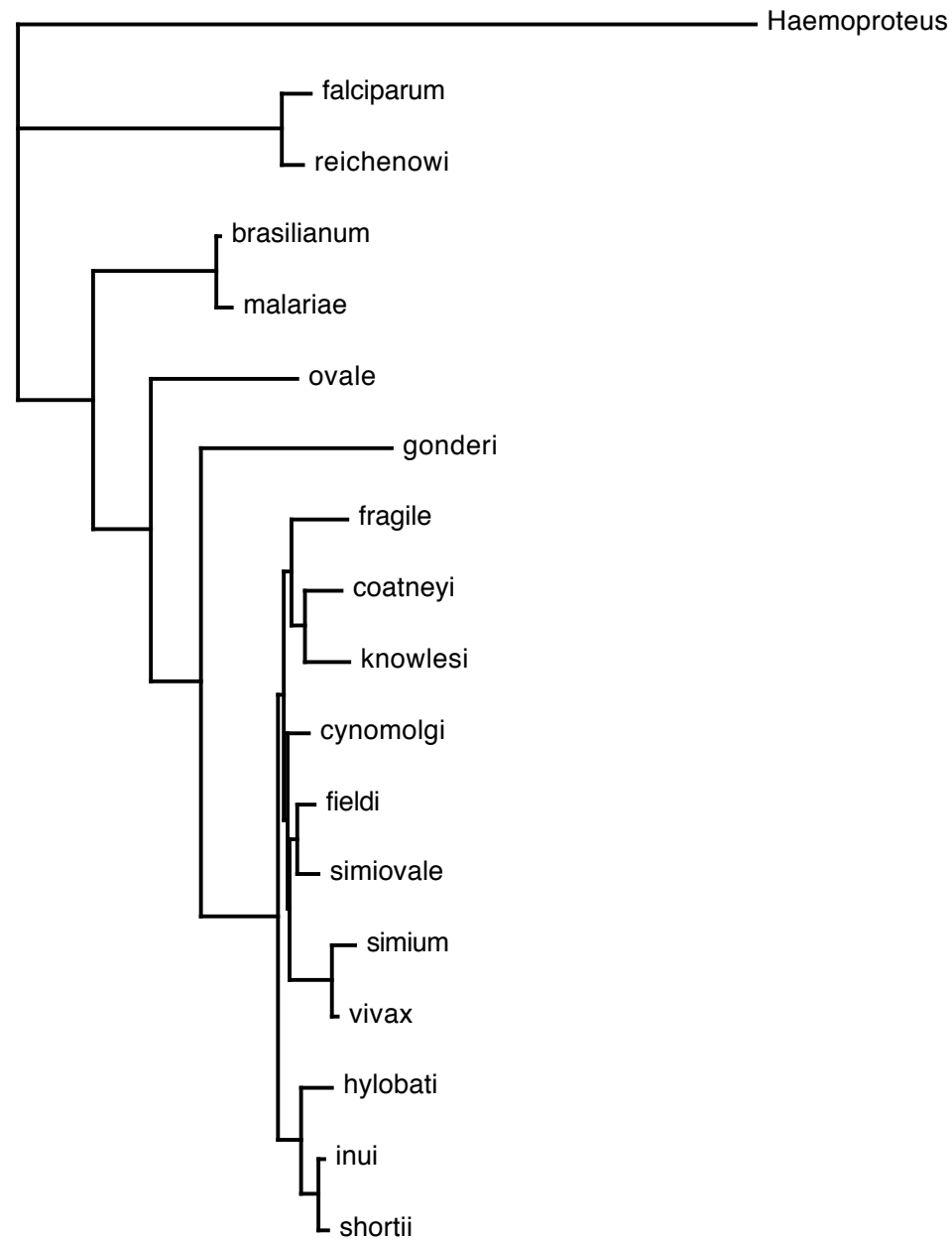

0.1

Supplement: Additional file 1 — Consensus phylogenetic hypotheses of Plasmodium parasites of primates from a Bayesian analysis of six genes. Consensus tree from a second modeling of phylogenetic relationships that excluded rodent parasites and Hepatocystis and provided alternative resolutions at the root. On the left panel, clade credibility trees are given with numbers at the nodes indicating the Bayesian posterior probabilities of each partition or clade in the tree, which are the proportion of trees in the sample that have the particular node. On the right panel, phylograms with branch lengths reflecting the expected substitutions per site is shown (see text for details). [file 1475-2875-8-110-S1.pdf]

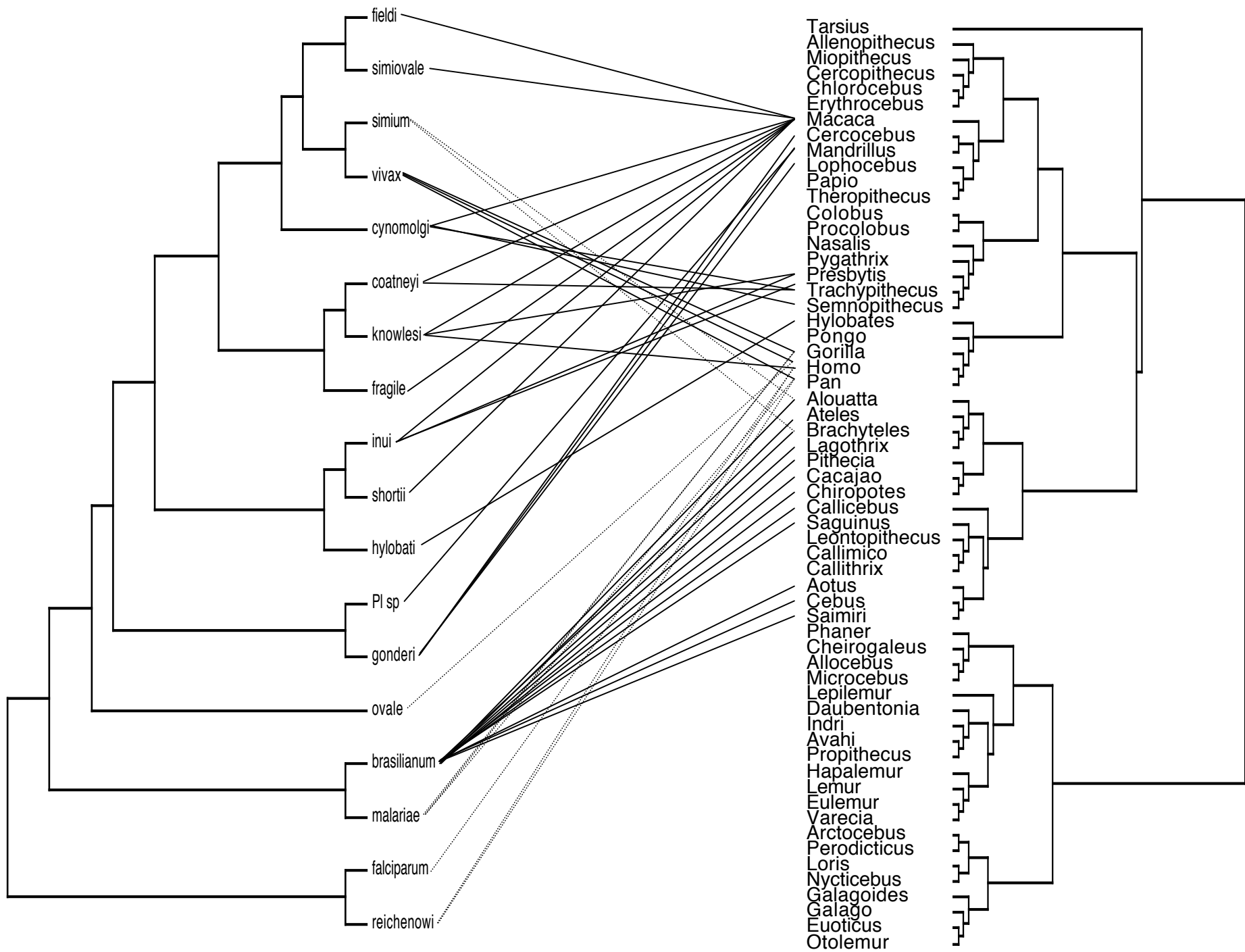

Supplement: Additional file 2 — The phylogenetic tree of the primate genera and their malaria parasite species. Parafit outcome when using the parasite phylogeny from Additional file 1. Connected taxa indicate naturally occurring infections. Solid lines represent host-parasite links that represent highly significant tendency for co-speciation, as shown by the ParaFit results. Dashed lines are for marginally (P ~0.1) significant relationships, while dotted lines indicate probabilities that correspond to random chance. Note that for simplicity, the phylogeny of hosts is not shown beyond the genus level, whereas the complete species-level phylogeny was used in the ParaFit analyses. [file 1475-2875-8-110-S2.pdf]

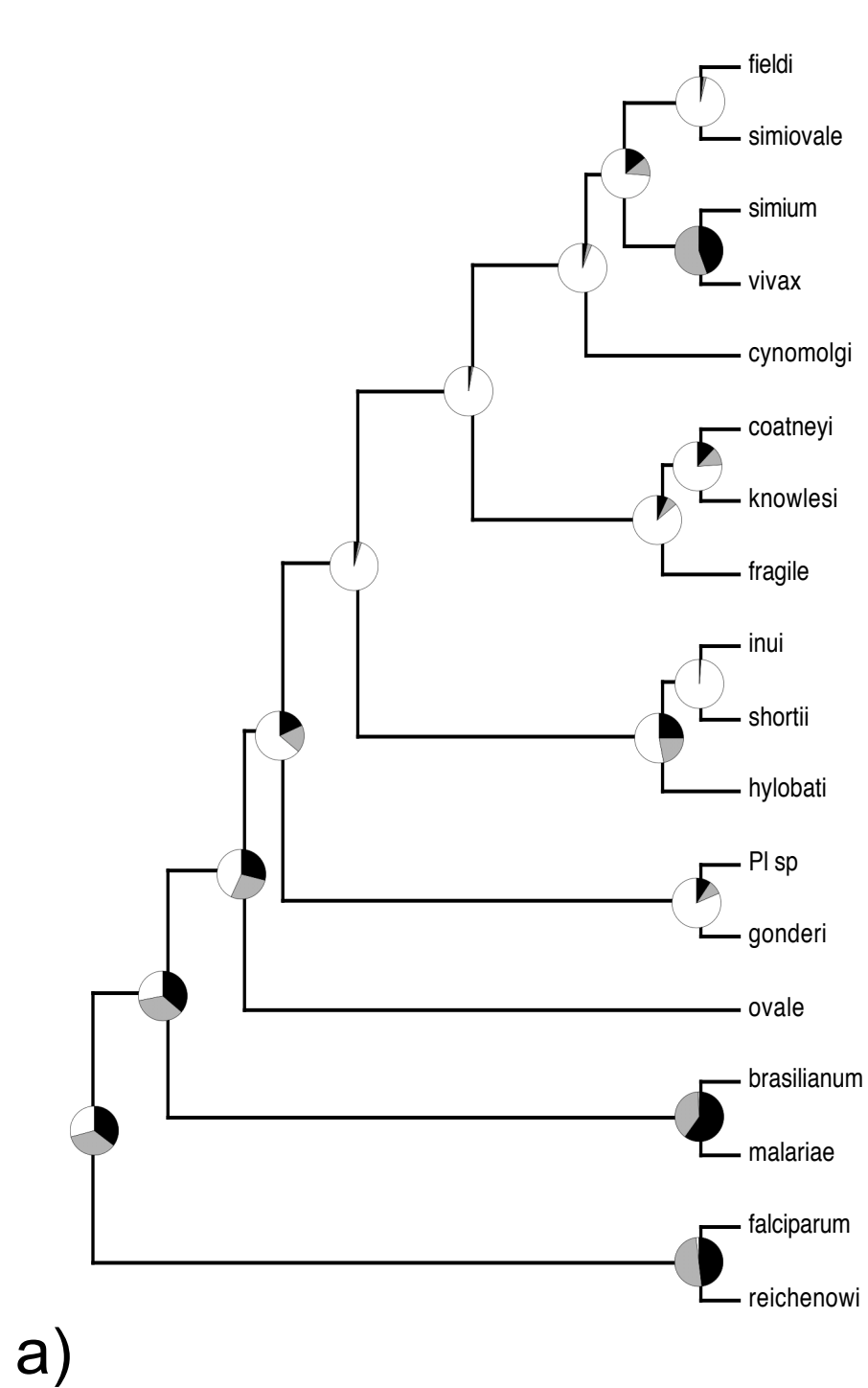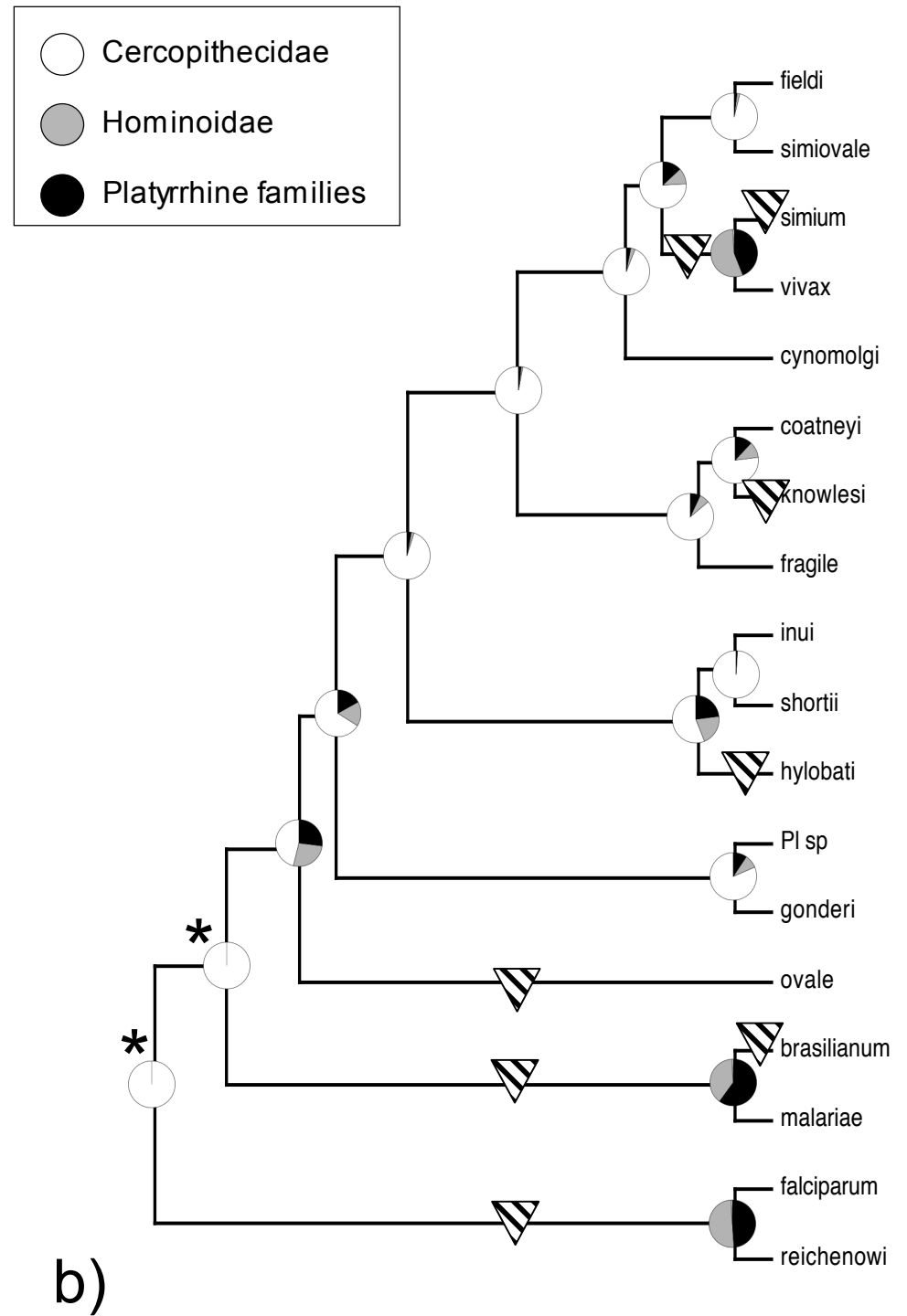

Supplement: Additional file 3 — Estimated ancestral states of host utilization based on Markov Chain Monte Carlo evolutionary modelling that used 1000 phylogenetic hypotheses. Circles summarize posterior densities of the reconstructed ancestral state from the Markov chain of 101,000,000 independent evolutionary models. Pie charts present probabilities of hosts being hominoid, cercopithecid or platyrrhine primate, respectively. a): Ancestral state estimations, when no restrictions were made, and each node was allowed to take any of the tree states. b): Estimations, when information on fossil records (Hominoids were unlikely to be present for parasites to infect around the origin of primate malarias) was used, and the two deepest nodes (marked with asterisk) were forced to have zero probability for hominoid host use. Results obtained when using the Bayesian sample of phylogenetic trees of parasites summarized on Additional file 1. Triangles show branches where host switch across large phylogenetic distances should have occurred. [file 1475-2875-8-110-S3.pdf]
